# Moscatilin suppresses the inflammation from macrophages and T cells

**DOI:** 10.1515/med-2022-0456

**Published:** 2022-04-13

**Authors:** Ying Zhang, Yugang Xu, Xiujie Jing, Wenkui Lu, Fusen Zhang, Chengkun Qin

**Affiliations:** Department of Hepatobiliary Surgery, Taian City Central Hospital, Taian 271000, Shandong, China; Department of General Surgery, Taian City Central Hospital, Taian 271000, Shandong, China; Department of Pediatrics, Taian City Central Hospital, Taian 271000, Shandong, China; Department of General Surgery, Dongping People’s Hospital, Dongping 271500, China; Department of Critical Care Unit, Taian City Central Hospital, Taian 271000, Shandong, China; Department of Hepatobiliary Surgery, Shandong Provincial Hospital, No. 324 Jingwuwei Road No.7, Jinan 250021, Shandong, China

**Keywords:** moscatilin, autoimmune liver disease, anti-inflammatory response

## Abstract

In this study, we aim to investigate moscatilin in alleviating symptoms of autoimmune liver disease (ALD) in a concanavalin A (ConA)-induced liver injury mouse model and elucidate the underlying mechanisms. ALD mouse models were constructed by intravenous injection of ConA (20 mg/kg) and the serum level of alanine aminotransferase (ALT) was measured using an enzyme-linked immunosorbent assay. Moscatilin in various doses was administered for two days starting from a day before the ConA injection. We showed that moscatilin dose-dependently decreased ALT levels in liver tissue of ALD mouse models. *Ifng* and *Tnfa* also showed significant downregulation in liver tissues. Macrophages only showed significant *Tnfa* downregulation and CD4^+^ T cells only showed significant *Ifng* downregulation at high moscatilin doses. *In vivo* administration of moscatilin induced interleukin-37 upregulation in hepatic tissues. *In vitro*, moscatilin also induced IL-37 upregulation in hepatic stellate cell line JS-1 rather than immune cells represented by RAW264.7 and CTLL-2 cell lines, suggesting that the hepatic stellate cell is majorly responsive to moscatilin treatment in terms of interleukin (IL)-37 upregulation. Our data indicate that moscatilin could alleviate liver injury in ConA-induced ALD mouse models through anti-inflammatory activities, warranting further development of moscatilin as a new drug in treating ALD.

## 1 Introduction

Autoimmune hepatitis (AIH) is a life-threatening disease caused by T cell-dependent liver injury [1]. Patients with AIH have a high risk of developing massive hepatic tissue damage and multiple-organ failure. AIH is characterized by aberrant production of proinflammatory cytokines including interferon (IFN)-γ and tumor necrosis factor (TNF)-α, which are factors inducing hepatic fibrosis and hepatocytes apoptosis [1,2,3,4]. Pharmacological approaches to mitigate immune dysfunction, particularly suppressing proinflammatory cytokine production, are highly desirable to improve treatment outcomes for patients with AIH.

Conventional drugs for treating autoimmune diseases include glucocorticoids, non-steroidal anti-inflammatory drugs, and analgesics [5]. Recently, herbal medicines and dietary natural products, which have been found to play crucial roles in maintaining human health [6], are increasingly being studied for treating autoimmune diseases [7,8,9,10]. Moscatilin [4-(4-hydroxy-3-methoxyphenethyl)-2,6-dimethoxyphenol] is a bibenzyl derivative extracted from Chinese traditional medicines including orchid *Dendrobium loddigesii* and *D. nobile *[11,12]. The known pharmacological capabilities of moscatilin include antioxidant, anti-inflammatory [11,13], anticancer [14], and antiplatelet aggregation effects [15]. Although it has not been explored whether moscatilin could be used for treating autoimmune diseases, the source of moscatilin, *Dendrobium,* was shown to be potentially valuable in the clinical management of diseases such as type 2 diabetes [16] and Sjögren’s syndrome [17]. Due to its anti-inflammatory role, moscatilin hypothetically could serve as an immunoregulatory drug to mitigate autoimmune diseases, where aberrant immune cells and cytokine production account for one of the major pathogenic factors.

In this study, we set out to evaluate the potential of moscatilin in alleviating concanavalin A (ConA)-induced AIH in mice. ConA is a plant-derived lectin known to stimulate mouse T-cells, which results in severe liver injury [18]. The injured hepatic tissues then promote the release of nitric oxide and reactive oxygen species, aggravating the injury. The ConA-induced liver injury mouse models recapitulate the pathogenesis in the human liver, in which we will investigate if moscatilin could suppress IFN-γ and TNF-α levels, in turn reducing liver damage induced by ConA [19]. We will also focus on evaluating whether moscatilin treatment can upregulate IL-37, a putative inhibitor of innate immunity by forming a complex with IL-18 receptor alpha and IL-1 receptor 8 to inhibit post-receptor signal transduction of pro-inflammatory genes [1,20]. Previous studies have suggested that IL-37, a natural inflammation modulator to alleviate malignant immune reactions, plays a critical role in promoting liver recovery in ConA-induced mouse models [1,21].

## 2 Materials and methods

### 2.1 Animals and treatment procedures

Balb/c mice (Male, 20 and 25 g, 7–9 weeks old) were acquired from GemPharmatech (Nanjing, China). All animal experiments were performed in compliance with protocols approved by Taian City Central Hospital. Moscatilin was solubilized in dimethyl sulfoxide. Before injection, moscatilin stock solution was dissolved in cremophor EL/ethanol and diluted in a 5% glucose solution. Moscatilin in various doses was intragastrically injected daily (days 0, 1, and 2). ConA (Merck Millipore) dissolved in saline was injected through the tail vein at the dose of 20 mg/kg on day 2 to induce liver injury [22]. For IL-37 blocking studies, mice were treated with both moscatilin and IL-37 neutralizing antibodies (R&D systems, Minneapolis, MN, USA, i.v., 0.4 g/kg/day on days 0, 1, and 2). Tissues were snap-frozen in liquid nitrogen and kept at −80°C until further analysis. During treatment, mice were allowed free access to water and food. Mice were sacrificed on day 3 using cervical dislocation and liver tissue and blood were collected.

### 2.2 Measurement of alanine aminotransferase levels

An enzyme-linked immunosorbent assay (ELISA) kit (Abcam, USA) was used to analyze the level of alanine aminotransferase (ALT) in mouse serum.

### 2.3 Quantitative real-time polymerase chain reaction (qRT-PCR)

Total RNA was isolated from frozen liver and then reversely transcribed into cDNA using a kit (TaKaRa Biotechnology, China) according to the manufacturer. qRT-PCR was performed using a 7900HT system (Applied Biosystems, USA), and the SYBR Premix EX Taq reagents (TaKaRa Biotechnology, China). The expression levels of the target gene were normalized to β-actin expression. The following primers were used: *Ifng* (IFN-γ), sense 5′-ATG AACGCTACACACTGCATC-3′, antisense 5′-CCATCCTTTTGCCAGTTCCTC-3′; *Tnfa* (TNF-α), sense 5′-CAGGCGGTGCC TATGTCTC-3′, antisense 5′-CGATCACCCCGAAGTTCAGTAG-3′; *Actb* (β-actin), sense 5′-AGGGAAATCGTGCGTGAC-3′, antisense 5′-CGCTCGTTGCCAATAGTG-3′.

### 2.4 Cell isolation, culture, and flow cytometry

Hepatocytes were isolated from hepatic tissues using the two-step collagenase perfusion technique [23]. Gradient centrifugation was used to isolate the macrophages and non-parenchymal cells from hepatocytes as described previously [24,25]. The CD4^+^ T cells were isolated from non-parenchymal cells labeled with phycoerythrin-conjugated anti-CD4 (eBioscience, USA) and sorted on the FACSAriaII cell sorter, (BD Bioscience, USA). Flow cytometry was also used to detect M1 (CD38+ Egr2−) macrophages and M2 (CD38–Egr2+) macrophages among CD11b+ cells. JS-1, RAW264.7, and CTLL-2 cell lines were acquired from American Type Culture Collection (ATCC, USA) and cultured according to recommended protocols. Cells seeded at 500,000 cells/well in six-well plates were treated with moscatilin of 1−50 µM for 48 h before RNA extraction for qRT-PCR analysis.

### 2.5 Western blotting analysis

Tissue protein was extracted using radioimmunoprecipitation lysis buffer supplemented with phenylmethane-sulfonyl fluoride and protease inhibitors. The bicinchoninic acid protein assay (Kaiji, China) was used to determine the protein concentration in the lysates. The proteins were resolved on sodium dodecyl sulfate gels and transferred onto nitrocellulose membranes. Membranes were blocked using 5% non-fat dried milk in phosphate-buffered saline (PBS). Then, membranes were incubated with primary antibodies overnight at 4°C. After being washed with PBS three times, membranes were incubated with the horseradish peroxidase-conjugated secondary antibody for 1 h. Subsequently, the membranes were washed three times, the electrochemiluminescence substrates (Optiblot, Abcam) were added and scanned with an imager (Abcam USA). Glyceraldehyde-3-phosphate dehydrogenase (GAPDH) antibody was from Abcam (ab8245) and IL-37 antibody was from Invitrogen (PA5-115405). GAPDH was used as the loading control.

### 2.6 Statistical analysis

The differences between the two groups were analyzed using one-way analysis of variance followed by a Tukey’s *post hoc* test. *p* < 0.05 was considered statistically significant.


**Ethics approval:** All animal experiments were performed in compliance with protocols approved by Taian City Central Hospital.

## 3 Results

### 3.1 Moscatilin alleviates liver damages

**Figure 1:**
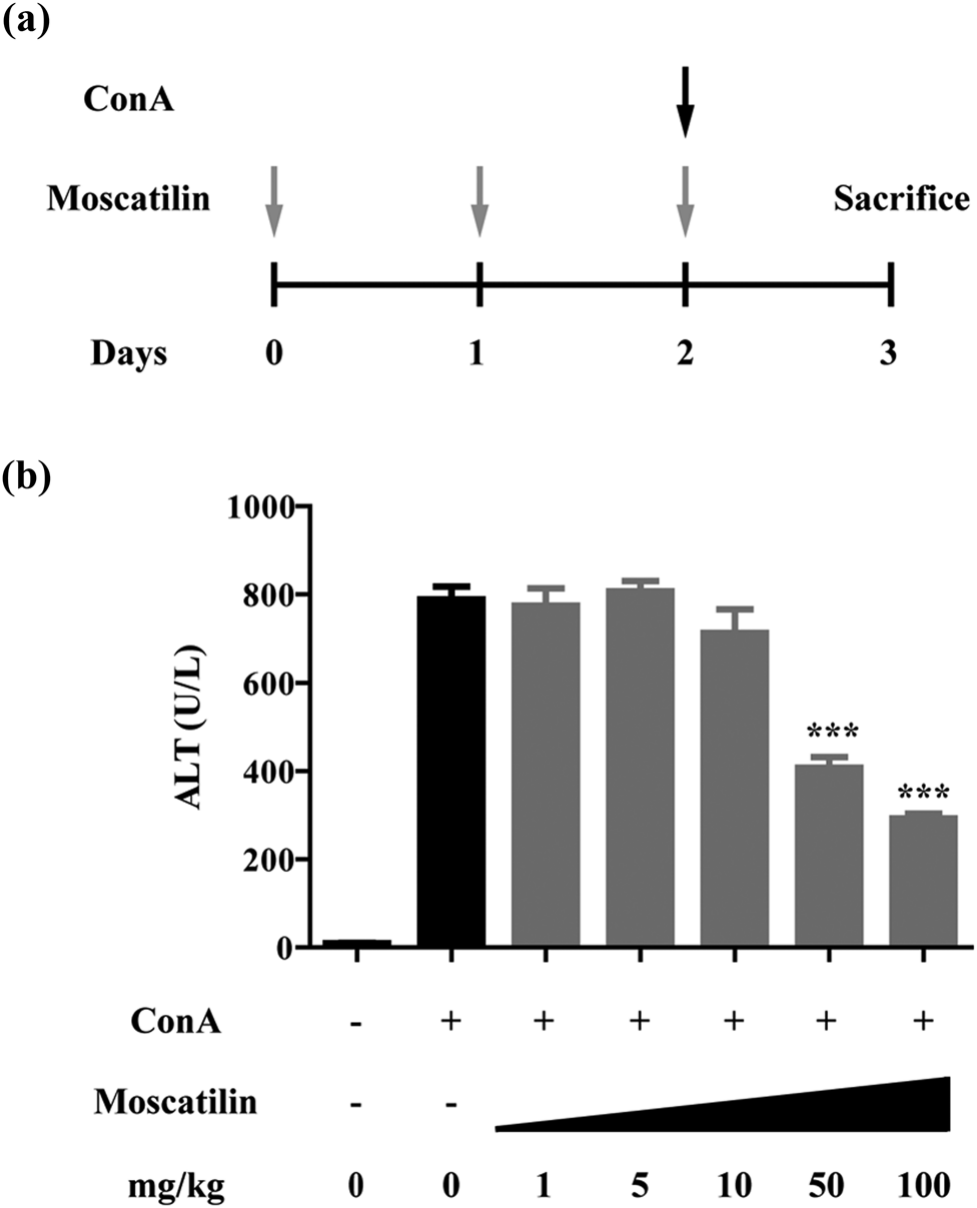
Moscatilin suppresses the ConA induced liver damage. (a) Schematic operation for the liver injury mice model. The mice received the different dose of moscatilin as indicated by intragastrical administration once a day and ConA (20 mg/kg) was intravenous injected in the 2nd day. (b) Serum level of ALT in the mice from different group at day 3 was detected by ELISA. Each group contained at least 10 mice and *** indicated *p* value less than 0.001 compared to the ConA group.

The scheme for our experiment is shown in Figure 1a. Mice were intravenously given moscatilin at a dose ranging from 1 to 100 mg/kg daily for two days. On the third day, moscatilin was administered together with ConA (20 mg/kg, intragastrically). After 24 h hours, mice were sacrificed and we collected blood and hepatic tissue. Mice treated only with ConA (negative control group) and normal mice (blank group) were used as controls. The serum levels of ALT were measured. Our data suggested that ConA treatment resulted in marked upregulation of ALT levels compared to the blank group, indicating liver injury, while moscatilin administered at high doses (50 and 100 mg/kg) was effective in downregulating ALT levels (*p* < 0.001 compared to ConA group, *N* = 10) (Figure 1b). Therefore, moscatilin exerted protective effects against liver injury in the ConA mouse models.

### 3.2 Moscatilin downregulates IFN-γ and TNF-α expression in injured liver

**Figure 2:**
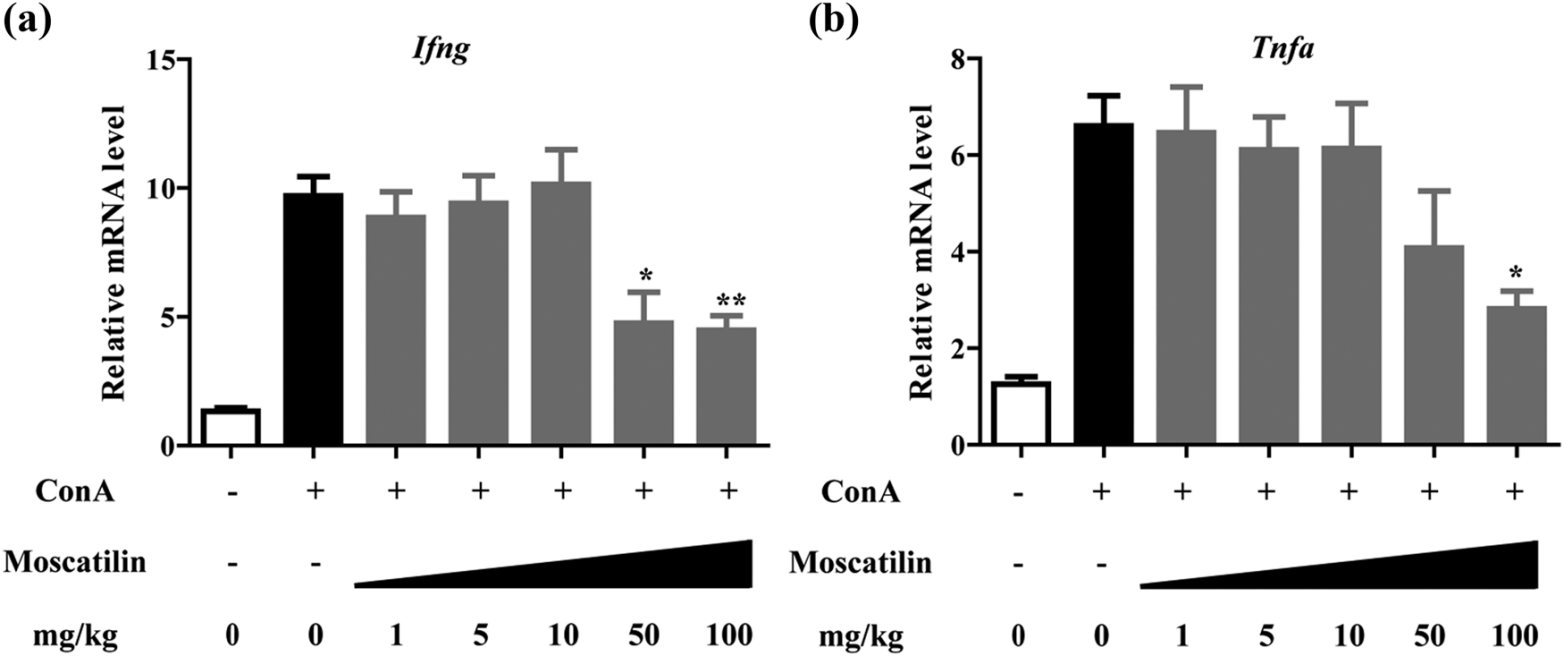
Moscatilin suppresses the hepatic production of IFN-γ and TNF-α. The mRNA expression of *Ifng* (a) and *Tnfa* (b) genes in the liver tissues was detected 24 h after the ConA injection by real-time RT-PCR. Each group contained five tissue samples and * indicated *p* value less than 0.05, ** indicated *p* value less than 0.01 compared to the ConA group.

We performed qRT-PCR analysis to examine the expression of *ifng* and *tnfa*, which encode two proinflammatory cytokines IFN-γ and TNF-α, respectively, in hepatic tissues. As shown in Figure 2a and b, in contrast to pronounced upregulation of *Ifng* and *Tnfa* in ConA mice, those treated with moscatilin showed significant downregulation of both *Ifng* and *Tnfa* (*p* < 0.05 for *Ifng* at the moscatilin dose of 50 mg/kg and *p* < 0.01 for *Ifng* at the dose of 100 mg/kg; *p* < 0.05 for *Tnfa* at the dose of 100 mg/kg; compared with ConA group). These data suggest the role of moscatilin in downregulating inflammatory factors.

### 3.3 Moscatilin suppresses the IFN-γ expression by CD4^+^ T cells and TNF-α expression by macrophages

**Figure 3:**
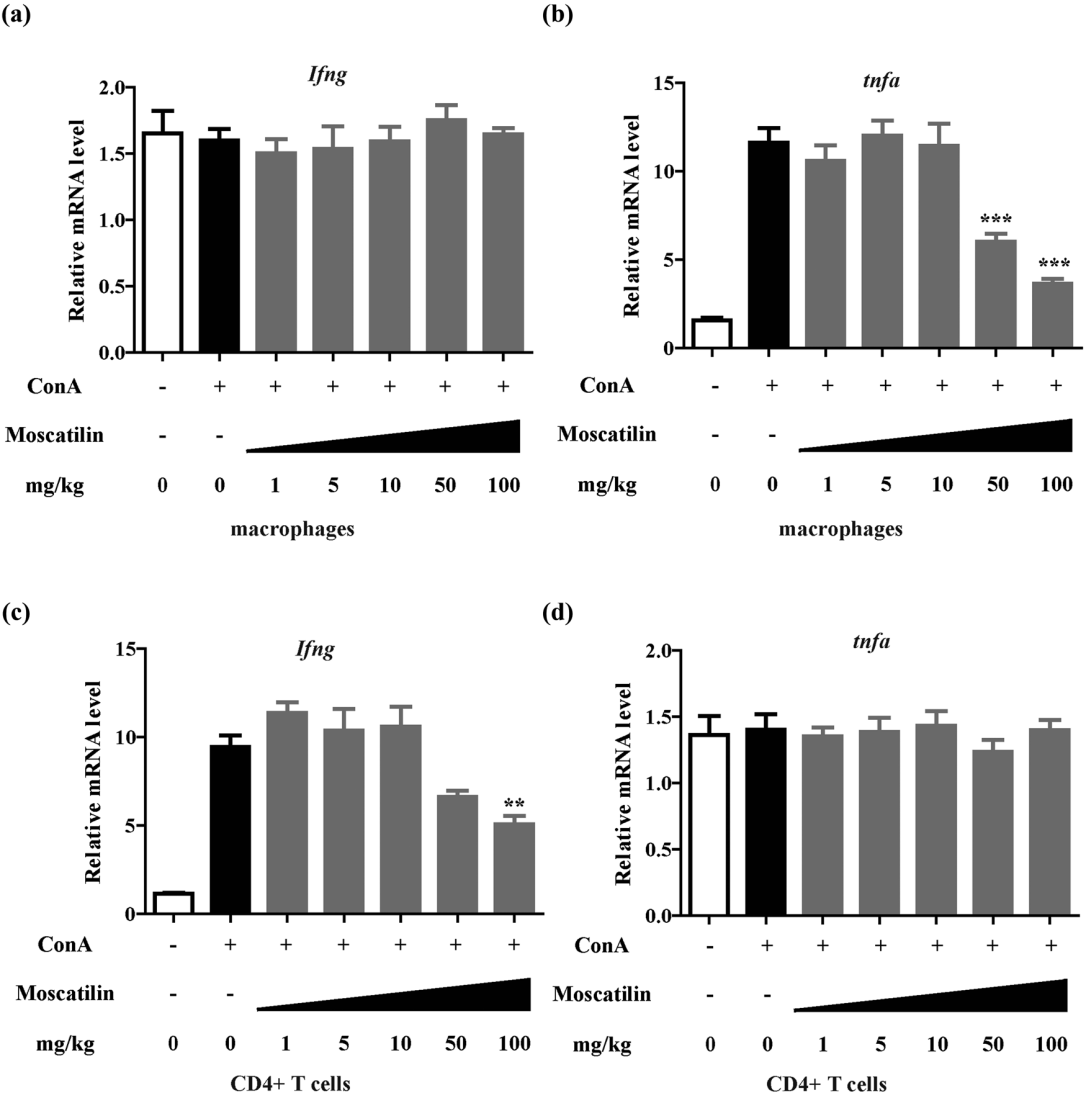
Moscatilin suppresses the IFN-γ expression from CD4^+^ T cells and TNF-α expression in macrophages. (a and b) Macrophages were isolated from liver tissues 24 h after the ConA injury and the mRNA expression of *Ifng* (a) and *Tnfa* (b) genes was detected by real-time RT-PCR. Each group contained 5 samples and *** indicated p value less than 0.001 compared to the ConA group. (c and d) CD4^+^ T cells were isolated from liver tissues 24 h after the ConA injury and the mRNA expression of *Ifng* (c) and *Tnfa* (d) genes was detected by real-time RT-PCR. Each group contained 5 samples and ** indicated *p* value less than 0.01 compared to the ConA group.

**Figure A1:**
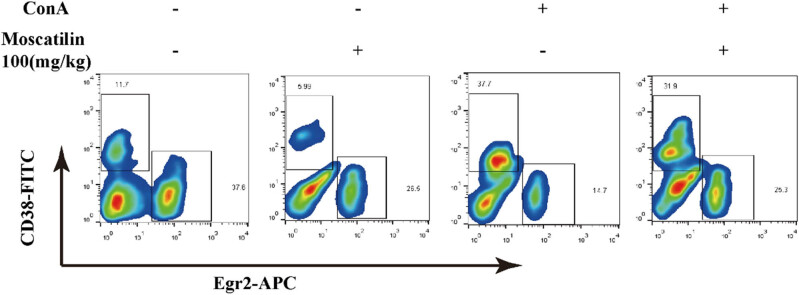
Effects of moscatilin on the M1/M2 macrophages. Macrophages in the liver tissues from the mice from different group at day 3 were gated in CD11b+ cells by flow cytometry. Then M1 macrophages were characterized by CD38+ Egr2− and M2 macrophages were characterized by CD38-Egr2+.

To elucidate the specific cells mostly affected by moscatilin treatment, we isolated macrophages and CD4^+^ T cells from hepatic tissues and analyzed *Ifng* and *Tnfa* expression. The population of CD4^+^ T cells was 9.57 ± 1.59% in ConA mice without moscatilin treatment and 8.92 ± 2.03% in mice with moscatilin treatment, respectively (*p* > 0.05). It turned out that *Ifng* expression in macrophages and *Tnfa* expression in CD4^+^ T cells were barely affected by moscatilin treatment (Figure 3a and d). Macrophages demonstrated the most prominent *Tnfa* downregulation after being treated with moscatilin at high doses (*p* < 0.001 at 50 and 100 mg/kg), while CD4^+^ T cells demonstrated the most prominent *Ifng* downregulation after being treated with moscatilin at 100 mg/kg (*p* < 0.01) (Figure 3b and c). The phenotypes of macrophages were also analyzed by flow cytometry analysis of M1 (CD38+ Egr2−) and M2 (CD38– Egr2+) macrophages, which showed that mice with or without moscatilin treatment showed no significant difference in M1/M2 macrophage phenotypes (Figure A1).

### 3.4 Moscatilin upregulates IL-37 expression in the hepatic tissues

**Figure 4:**
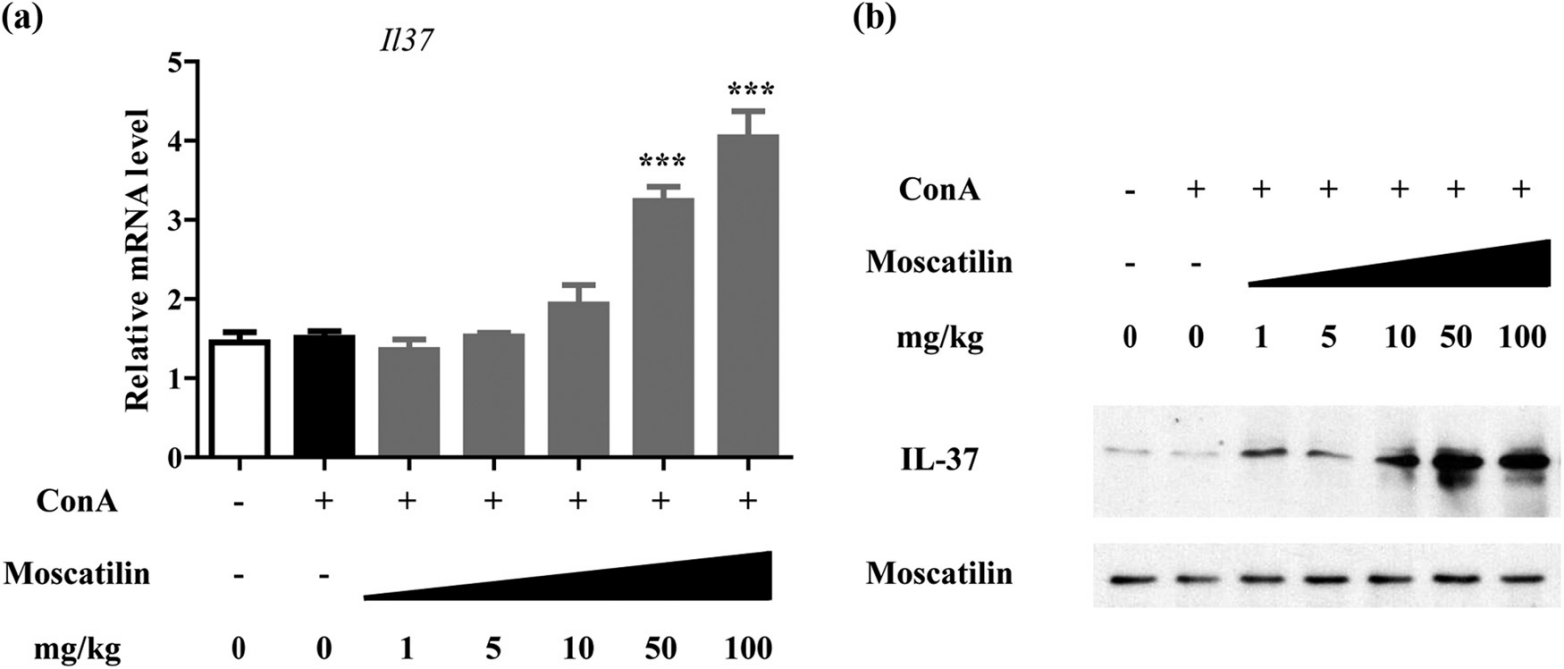
Moscatilin induced IL-37 expression in the hepatic tissues. (a) The mRNA expression of *IL37* genes in the liver tissues was detected 24 h after the ConA injection by real-time RT-PCR. Each group contained 5 tissue samples and *** indicated *p* value less than 0.001 compared to the ConA group. (b) The protein level of IL-37 in the liver tissues was detected 24 h after the ConA injection by western blot. GAPDH was used as the loading control.

**Figure A2:**
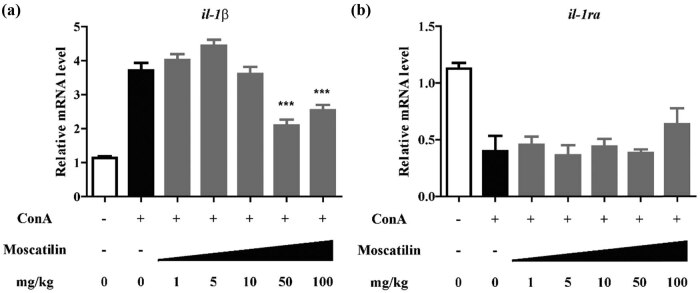
Effects of moscatilin treatment on the hepatic production of IL-1β and IL-1Ra. The mRNA expression of *il-1β* (A) and *il-1ra* (B) genes in the liver tissues was detected 24h after the ConA injection by real-time RT-PCR. Each group contained 5 tissue samples and *** indicated p value less than 0.001 compared to the ConA group.

**Figure A3:**
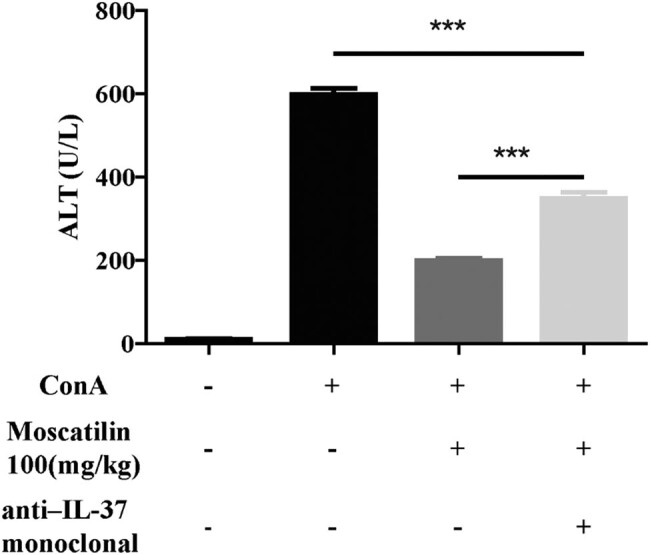
Blocking IL-37 could reverse the effects of moscatilin on the ConA induced liver damage. Serum level of alanine aminotransferase in the mice from different group at day 3 was detected by ELISA. Each group contained at least 10 mice and *** indicated *p* value less than 0.001 for comparation indicated.

We next analyzed the expression level of the anti-inflammatory cytokines including IL-1β, IL-1Ra, and IL-37 in hepatic tissue (Figure A2). Expectedly, moscatilin increased dose-dependent IL-37 expression on both the mRNA level (*p* < 0.001 at 50 and 100 mg/kg, Figure 4a) and protein level (Figure 4b), suggesting the anti-inflammatory effects of moscatilin. IL-1β (Figure A2a) but not IL-1Ra (Figure A2b) demonstrated responsiveness to moscatilin treatment. To confirm that IL-37 plays a dispensable role in the therapeutic effects of moscatilin, we treated mice simultaneously with intravenous injection of IL-37 neutralizing antibodies. As shown in Figure A3, ALT levels were higher in mice treated with both moscatilin and IL-37 neutralizing antibodies, suggesting abrogated the injury alleviation by moscatilin in Con-A mice (Figure A3).

### 3.5 Moscatilin upregulates IL-37 expression in the hepatic cells *in vitro*

**Figure 5:**
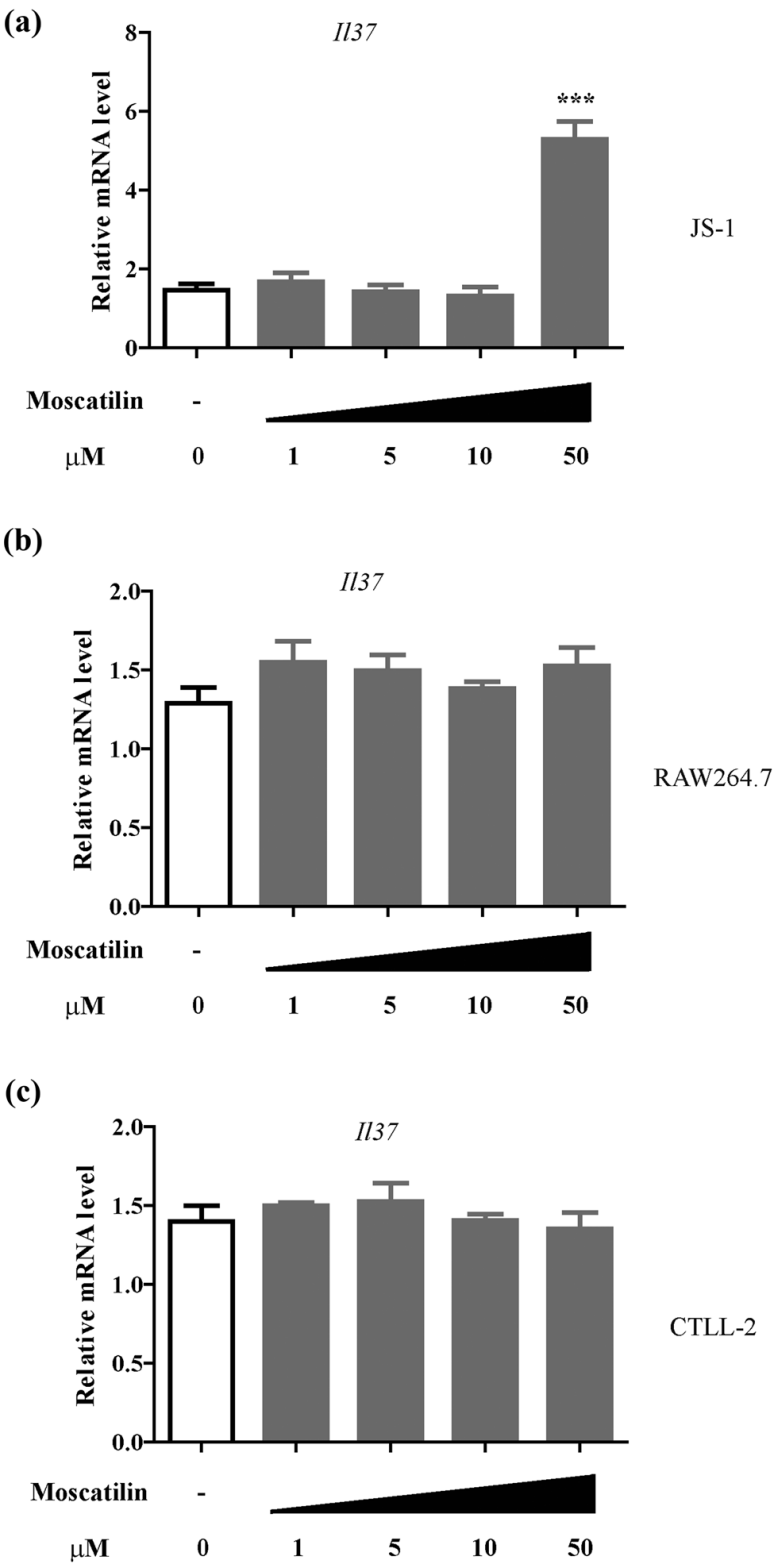
Moscatilin induced IL-37 expression in the hepatic cells *in vitro*. Hepatic stellate cell line (JS-1) (a), macrophage cell line (RAW264.7) (b) and T cell line (CTLL-2) (c) were treated with different dose of moscatilin for 48 h and the mRNA level of Il37 gene was detected by real-time RT-PCR. *N* = 5 for each group. *** indicated *p* value less than 0.001 compared to control group.

**Figure A4:**
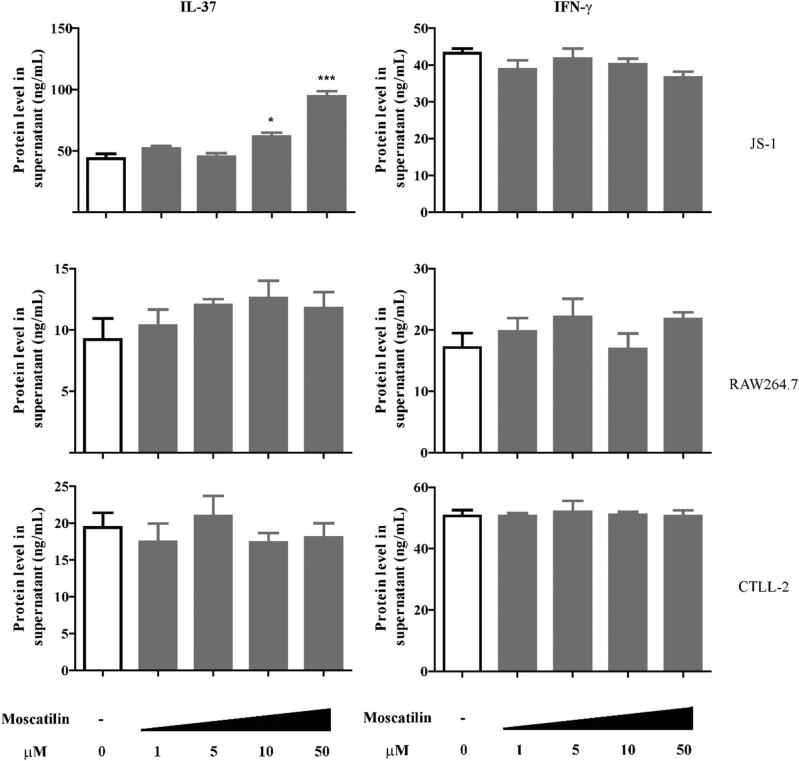
Effects of moscatilin treatment on IL-37 and IFN-γ protein level in the cells in vitro. Hepatic stellate cell line (JS-1), macrophage cell line (RAW264.7) and T cell line (CTLL-2) were treated with different dose of moscatilin for 48 hours and the protein level of IL-37 (left) and IFN-γ (right) in the supernatant was detected by ELISA. Each group contained 5 samples and * indicated p value less than 0.05, *** indicated *p* value less than 0.001 compared to control group.

To further clarify the cell source of IL-37 upregulation, we cultured hepatic stellate cells (JS-1), macrophages (RAW264.7), and T cells (CTLL-2) *in vitro* in presence of moscatilin. It turned out among cells tested, only JS-1 showed significant IL-37 upregulation (*p* < 0.001 at 50 μM, Figure 5a–c), indicating that hepatic stellate cells may be a major type of cells responding to moscatilin treatment in terms of IL-37 production. The secreted IL-37 and IFNG levels were also analyzed by ELISA assay (Figure A4), which showed that the level of secreted IL-37 but not IFNG is responsive to moscatilin treatment.

## 4 Discussion

Here we performed a preclinical study to investigate the potential of moscatilin in alleviating autoimmune liver disease (ALD). The pharmacological properties of this herb-derived compound have been previously explored with respect to anti-cancer, anti-inflammatory, and antioxidant activities, but its utility in countering autoimmune diseases has not been explored. We aimed to expand the utility of moscatilin for treating ALD, in which excessive production of proinflammatory cytokines leads to severe hepatic tissue injury. This new application of moscatilin is in line with its immunoregulatory role reported in our previous studies [13,26]. We exploited a ConA-induced liver injury model and for the first time showed the efficacy of moscatilin in alleviating liver injury in the preclinical setting. Concomitant with ALT level reduction, hepatic production of the major proinflammatory cytokines, that is, IFN-γ and TNF-α, were shown to be significantly downregulated at a moscatilin dose of 50 and 100 mg/kg. It is worth noting that treatment with moscatilin was initiated two days prior to ConA administration; therefore, our data is limited to demonstrating the protective effect of moscatilin against liver injury. Further study is warranted to explore the injury-alleviating efficacy of moscatilin when the injury has already occurred.

We next examined which types of immune cells in the liver were most responsive to moscatilin treatment in terms of IFN-γ and TNF-α downregulation. We showed that IFN-γ downregulation is only prominent in CD4^+^ T cells while TNF-α downregulation is only prominent in macrophages. These findings were in agreement with the notion that IFN-γ and TNF-α were mainly produced by T cells and macrophages respectively. M1 macrophages serve as the main type of cells to produce hepatic TNF-α [27,28,29]. The reduction in TNF-α may suggest a transition of M1 macrophages (pro-inflammatory) to M2 macrophages (anti-inflammatory) [30,31,32] as M2 macrophages are less efficient in TNF-α production [33,34]. The suppression of IFN-γ expression by T cells may also be a result of reduced M1 macrophages, which leads to diminished production of IL-12 and IL-1β that promote IFN-γ production by T cells. On the other hand, M2 macrophages produce IL-10 and IL-1Ra that suppress IFN-γ production by T cells. Macrophages and T cells have been used as major cell targets for treating liver injury in ConA mice. For example, strategies to deplete macrophages have been shown to suppress the induction of hepatitis [35,36]. Bone marrow-derived mesenchymal stem cells, with the capability of switching the phenotypes of macrophage from M1 to M2, have been shown to attenuate ConA-induced liver injury [37]. Approaches to increase the M2 macrophages and suppress the polarization of macrophages to M1 were also demonstrated effective in alleviating liver injury [38]. An anti-CD4 monoclonal antibody that antagonizes CD4^+^ T cells can protect against liver damage [35]. Our data suggest that moscatilin treatment serves as another pharmacological approach to modulate macrophages and T cells and thereby has significant potential in regulating cytokine production in the injured liver.

Given that IL-37 is a natural inflammation modulator [39], we also focused on evaluating how moscatilin affected IL-37 expression in ConA-treated mice. Indeed, IL-37 was upregulated in hepatic tissues on both mRNA and protein levels, therefore synergizing with its effects in suppressing proinflammatory cytokines. As suggested by previous studies, IL-37 is also an important regulator of the intrahepatic production of IFN-γ and TNF-α, and efforts have been devoted to overexpressing or administering exogenous IL-37 to assess how it affected T cell-dependent liver injury [40,41]. Besides, we also clarified the type of cells that is responsible for IL-37 upregulation upon moscatilin treatment. Among hepatic stellate cells, macrophages, and T cells treated with moscatilin *in vitro*, only the hepatic stellate cells showed significant upregulation of IL-37. This observation appeared to contradict our findings *in vivo* that moscatilin regulates cytokine expression by macrophages. We postulated that although moscatilin treatment of RAW264.7 and CTLL-2 cells *in vitro* did not affect IL-37 expression, the pharmacokinetics of moscatilin treatment *in vivo* can be different. For example, hepatic stellate cells can affect immune cells by paracrine secretion. The mechanism remains to be further elucidated. Taken together, these data shed light on the biological responses in the liver following moscatilin treatment, including changes in cytokine levels and exact cell types responsible for the changes, which could serve as foundations for further evaluation of moscatilin in other animal models and ultimately human, as well as elucidating molecular underpinning of moscatilin in treating ALD.

## 5 Conclusion

Our findings indicated that moscatilin is effective in alleviating hepatic injury in ConA-treated mice, supported by the significant downregulation of serum ALT levels, hepatic IFN-γ, and TNF-α levels, and increased IL-37 levels. Downregulation of IFN-γ and TNF-α was most prominent in CD4^+^ T cells and macrophages, respectively and hepatic stellate cells were responsible for the observed upregulation of IL-37. Our data, therefore, support that moscatilin is a potential new drug for treating ALD and further development.
